# Validation of a Rubric to Evaluate Open Educational Resources for Learning

**DOI:** 10.3390/bs9120126

**Published:** 2019-11-25

**Authors:** Anabel de la Rosa Gómez, José Manuel Meza Cano, Germán Alejandro Miranda Díaz

**Affiliations:** Faculty of Higher Studies Iztacala, National Autonomous University of Mexico, Mexico City 04510, Mexico; manuel.meza@iztacala.unam.mx (J.M.M.C.); amiranda@iztacala.unam.mx (G.A.M.D.)

**Keywords:** Open Educational Resources, rubric, validation

## Abstract

Open Educational Resources (OERs) include different web formats, such as audio, video, images and text that can be modified, redistributed and used for learning about a specific topic, however, it became pertinent to create an OER evaluation tool with content validity. The present work gives an account of the content validation process using a 41-criteria rubric, each one with three performance levels, developed from a documentary search on relevant aspects to be included in a learning object. Six expert judges were contacted and decided whether each criterion was essential, useful but not essential or not necessary. Based on this, the Content Validity Reason (CVR) was calculated, obtaining 32 validated criteria. Among some conclusions, it can be mentioned that the validation process of contents allowed the identification of those criteria that require modifications or, if necessary, can be discarded to increase the validity of the heading in its whole.

## 1. Introduction

The Open Educational Resources (OERs) are digital materials with the characteristic of being accessible for educators and users who wish to use or reuse them to learn about a specific topic, for research or teaching. The characteristic of being open allows the freedom to use them, study the content and apply the knowledge acquired through them, in addition to redistributing them and making changes or improvements [1].

The creation of educational resources and materials raises an important question about the relevance to the target population, their content and the way in which they are distributed. Some authors [2] mention that there are elements that must be taken care of to communicate the information and therefore generate in the student the expected improvement in the learning processes, always taking into account quality criteria in the OERs for their development.

Among the advantages of using OERs in education, it has been mentioned [3] that they have a positive impact on student learning when they are used to study while facing diverse learning situations, for example, prior to a summative evaluation. Nevertheless, it is necessary to care for important quality criteria for their development, dissemination and implementation.

When an OER is perceived as having poor quality in its elaboration or content, it may result in a barrier for its approval and usage [4], so it is recommended for the OER to be performed by a reputable institution with access to updated information, as well as to take care of technical aspects, such as ease of access or download, freedom to adapt and use, in addition to indicating the type of licensing.

For the type of input that an OER represents, since it contains multiple elements and dimensions at a technological and pedagogical level, the use of an analytical rubric is proposed to address the basic elements of its development, which serve as evaluation criteria. A rubric of this type takes into account various elements with their respective performance gradients and allows them to have an integral assessment, in addition to having basic characteristics such as clarity, specificity and objectivity [5].

By using a rubric, the evaluator makes judgments based on descriptive facts instead of assumptions, therefore the development of explicit evaluation criteria and their integration into rubrics in practice help to facilitate greater commitment and understanding about the action of evaluating performance in a specific domain [6]. In a general sense, a rubric is a matrix of performance evaluation in activities that are complex, based on a set of measured criteria that qualifies progressively the transit of a performance, evidencing a competent level in terms of the manifestation of elements considered essential [7].

The reasons for using rubrics are based on five ideas [8]: short response time for feedback, students’ preparation for detailed use of feedback, to foster critical thinking, to facilitate communication with others and to help perfect teaching techniques. Among their main features, they highlight [9] evaluation criteria, quality definitions for these criteria at particular levels and a punctuation strategy.

Recently, the rubrics have been used as instruments and techniques for the evaluation by competences. Currently and in the presence of new scenarios offered by technology, the rubrics have moved from paper into an electronic and interactive format constituting electronic rubrics, also known as e-rubrics [10]. An example of using a rubric to evaluate the implementation of the OER [11] integrates institutional elements, such as the infrastructure for the dissemination of the OERs, the coincidence of the OERs with the institution objectives and the integration of the OER with the curriculum of the official subjects. Nevertheless, an approximation of fine grain on the constituent elements of an OER from its structure is necessary.

Something important to point out is the problem of using rubrics without taking care of the systematic process of their elaboration [12], the reason why they are being used as an instrument to evaluate the performance in a task and for what activity or learning product their validity and reliability properties should be considered to be effective [13,14,15].

In a meta-analysis using 63 studies that addressed the rubrics and the validity process [16], it was found useful to distinguish between several types of evidence that can be applied to an argument to validate rubrics. When talking about content validity, the documentation source of the rubric content and the review by experts of this content are included. The criterion validity test usually includes correlations between rubric scores with external judgments from the same area or domain using other data sources. In internal validity tests, the relationships between the criteria within a rubric are often demonstrated using factor analysis to decide how the creation of dimensions can measure the construct better.

A study on criterion validity [17] employed statistical analyses, such as t-student and correlations, to relate the scores of a rubric with changes in variables related to the instruction in tasks on the development of writing skills in a pretest–posttest design, for which they took the results as evidence of validity. In another study [18], they related scores of a group of students in an academic writing assignment with the grades of teachers. By obtaining strong correlations between teacher qualifications and students, they determined that the rubric was sufficiently valid.

The intention of creating a rubric to evaluate an OER is to provide data for its subsequent improvement, for which it is interesting to develop the rubric with evidence of validity from its content. In this case, the criterion of expert judges is used. The criterion of judges technique is one in which, with the help of qualified and competent judges, the experts evaluate the content and determine whether they agree with the construct’s proposals, approving or disapproving the content. It is the most commonly used strategy to evaluate content validity [19]. The expert judgment is an informed opinion from people with experience in the subject, recognized by others as experts who can provide information, evidence, judgments and assessments. Authors affirm [20] that when they’re dealing with OERs, the review of the experts may be an uneconomical approach, but it seems to be necessary to evaluate the content of the objects that are integrated in an institutional repository.

Following the technique of expert judgment, the Content Validity Reason (CVR) has been used [21] in different studies: as a way to validate the items in various scales, for example, to validate the items of a scale in mathematical knowledge [22] or to validate the criteria of a registry that allowed the pedagogical monitoring of teachers in an institution [23]; to validate the items of an instrument to measure communication focused on patients in a hospital [24]; or to validate items on a scale to measure depression in geriatric patients [25]. Once this background has been reviewed, it is considered necessary to build a rubric to evaluate the OER, based on the validation of their criteria. Therefore, the objective of the present study is to describe the process of the creation and validation of the content of the criteria of a rubric to evaluate OERs.

## 2. Methods

### 2.1. Phase 1: Construction of the Rubric to Assess OER

From a documentary search, the necessary information was obtained for the elaboration of necessary criteria to evaluate OER, based on research works considered by the quality guidelines that mention [26,27,28,29,30,31].

From the review of this information, 41 criteria were identified, which cover different dimensions related to: the intention of learning didactics, motivational aspects, the use of instructional design, the clarity of written language, the grammar of language, the use of inclusive language, the function of the icons, interface clarity, navigability, hypertextuality, graphic design, the use of multimedia, the portability of objects, accessibility, licensing, among others.

Each of the criteria had three levels of performance: “incipient”, “in process” or “consolidated”. For example, for the “portability” criterion, the following three performance levels were developed as shown in Table 1. Later it was adapted to a Google form.

### 2.2. Phase 2: Validation of Criteria by the Panel of Experts

Six expert judges were selected based on the following criteria: (a) professionals who are engaged in online learning; (b) three years of experience in instructional design models applied to the development of open educational resources (OERs). They received the Google form by email with the rubric criteria as a panel for the evaluation of content [21]. The experts could indicate for each criterion whether it was essential, useful but not essential or not necessary, in addition to adding comments in an open space in the form.

Once the answers of each judge were obtained for each criterion of the rubric, the validity proportion was calculated with the following formula:

$$CVR=\frac{ne-\frac{N}{2}}{\frac{N}{2}}$$

where *ne* is the number of panelists that indicate that a criterion is “essential”. *N* is the total number of panelists.

When less than half of the panelists indicate that it is not essential, then the proportion is negative. When half of the panelists indicate that it is essential and the other half does not, the proportion is zero. When all the panelists indicate that the criterion is essential, then the proportion is one. When more than half of the panelists indicate that it is essential, but not all, then CVR is a value between zero and 0.99 [21]. Following the recommendations of this kind of study [31], the Item-Index of Total Content Validity (CVI) (number of agreements in “essential”/number of panelist) and the modified Kappa statistic were obtained and were added to the analysis by item.

## 3. Results

Below are the criteria for the heading, the CVR, I-CVI and Kappa of each item according to the expert panel. 

Criteria with CVR = 1, I-CVI = 1, K = 1, Excellent:

- C1—Intention of learning (didactic congruence);
- C2—Motivational aspects (maintains attention);
- C4—Written language (clarity);
- C9—Function of icons;
- C14—Hypertextuality (links according to the content);
- C17—Consistency of objectives (with content);
- C18—Graphic design (colors that allow reading);
- C20—Graphic design (organization between graphics-text);
- C21—Homogeneous graphic design (colors, logos, typography);
- C37—Portability (accessible from different devices);
- C39—Bibliographic references (from sources and images);
- C40—Author(s), institution and contact.

Criteria with CVR = 0.66, I-CVI = 0.83, K = 0.81, Excellent:

- C10—Interface (fluency and visualization);
- C11—Navigability (links according to content);
- C12—Hypertextuality (relevance in the text and its quantity);
- C22—Multimedia (didactic intention);
- C24—Images (didactic intention);
- C25—Images (size);
- C26—Images (good definition for viewing);
- C27—Audio (clarity, no interference);
- C32—Video (good size for viewing);
- C34—Video (didactic intention);
- C36—Video (quality of resolution);
- C41—Free access (license allows re-use, distribution).

Criteria with CVR = 0.33, I-CVI = 0.66, K= 0.57, Fair:

- C3—Instructional Design;
- C6—Adaptation of language to the population;
- C13—Hypertextuality (links open in new windows);
- C16—Hypertextuality (links functionality);
- C29—Audio (volume control);
- C30—Audio (control, pause, repeat);
- C35—Video (control by the user).

Criteria with less than zero CVR were considered Poor and discarded:

Criteria with CVR = 0, Poor:

- C5—Clarification of ideas (examples);
- C8—Inclusive language (non-sexist or racist);
- C15—Hypertextuality (easy identification of links);
- C19—Graphic design (fonts 14 pts or greater);
- C23—Multimedia (visual, auditory and textual);
- C33—Video (duration less than 4 min).

Criteria with CVR = −0.33, discarded:

- C7—Language grammar (passive voice);
- C28—Audio (duration, less than 4 min);
- C31—Audio (design, effects and curtains).

As can be seen, of the 41 criteria, 12 criteria reached a CVR of 1, which, according to Lawshe, is the minimum necessary to keep them within the scale, and 32 were obtained with a CVR score greater than zero. Therefore, nine criteria obtained zero or negative scores. Also, when calculating the Index of Total Content Validity (CVI) of the instrument from the mean of all the criteria, discarding the nine criteria with zero and negative scores, the CVI amounted to 0.708. Taking into account the 12 criteria with CVR = 1 then, logically, the CVI = 1 as well. To give an account of the comments about the criteria with a CVR equal to zero or negative, Table 2 was made.

There are aspects in the comments related to the relevance of the criteria, being in some cases valued as “not necessary” by the judges. This is the case of criterion 8 on the use of “inclusive language”, which received comments related to its little relevance and relevance in the rubric. There were suggestions on the presentation of the criteria in terms of the wording and the elements found in them (criterion 7).

## 4. Discussion

The process by which the rubric was structured required an attentive analysis of the sources. In addition, the theoretical justification for each of the criteria was considered important and included. Having an analytical rubric [5] of this type could reduce the possibility of evaluator’s bias as they are able to use it even when they have little experience in the development of OERs, because in the body of this instrument are the minimum essential elements [7]. This is important since authors [32] state that the results of the use of rubrics may vary due to the perception of their use by the evaluators.

Something important to highlight is that the use of the CVR, I-CVI and Kappa [21,24] allowed evaluators to discriminate between those criteria that judges considered non-essential to those that were, including a gradient of possibilities between both. Of the findings in the present investigation, nine criteria had a CVR of zero or negative. However, in other investigations [23], they took only counts of criteria with a CVR greater than 0.60 (I-CVI = 0.83), achieving a high coefficient of CVI in the complete instrument. These guidelines can serve as a basis for retaking only certain criteria of the heading and initiating the reliability process in a second moment.

Upon returning to the criteria discarded due to a low CVR, different actions were found at their execution. For example, criterion 33, “video duration”, did not obtain favorable consensus from the judges. One of the judges suggested a duration between 6 and 12 min. However, a review of the literature [33] found that they must meet a duration of between 5 to 10 min. Other authors mention that the design of videos as support for video tutorials should be under some didactic and technical considerations, among which is the plan to have a duration of between 10 and 20 min [34,35]. Other authors [36] recommend having a duration between 5 and 10 min, proposing that the user dedicate between 1 and 2 h a week to watching the videos.

In another example, related to criterion 8 “Inclusive Language”, the judges were divided between “essential” and “useful but not essential”. In this regard, it has been mentioned [31] that it is important to recognize the use of the OER to favor situations of equity through education, especially at a cultural level, respecting diversity at the same time, so it is important to make the decision to adapt the criteria and take these guidelines into account or discard them given their CVR, I-CVI and Kappa low coefficient.

Based on the findings of this study, the possibility of using the rubric with the validated criteria in the OER evaluation was proposed, so that they can be shared with the community, which would have the minimum essential elements that experts have determined. Likewise, the procedure followed in this study has made it clear that there are basic characteristics that an OER must contain, where in addition to aspects related to the use of multimedia and work in graphic design, educational elements that emphasize instructional design, such as the didactic intention, the clarity of the language used and the care of the motivational aspects, are essential, which moves away from the technological vision of the OER and focuses it on an educational emphasis. Carrying out the validity process allows the obtainment of data to refine the rubric and make decisions on the adaptation of the criteria or their elimination, in a continual effort to improve in this way so it can be used, taking into account the empirical support. Subsequently, we will continue with the recommendations of the literature [14] and the reliability process, using methods, such as the agreement percentage between applicators, or the use of statistics, such as the intraclass correlation coefficient [15].

## Figures and Tables

**Table 1:** Example of “portability” criteria and performance levels.

	Level 1. Incipient	Level 2. In Development	Level 3. Consolidated
Portability	Can only be displayed on a device with a specific program.	It is only displayed on two of the three devices (computer, tablets and smartphone).	The material can be seen from different devices (computer, tablets and smartphone).

Each of the 41 criteria had all three performance levels.

**Table 2:** Criteria discarded by low Content Validity Reason (CVR) and comments of the judges.

Num	Criterion	CVR	Comments
5	Clarification of ideas	0	More than clarification of ideas would be “exemplification of ideas”. “The qualification of ideas is only given by example?” “It varies its importance depending on the theme, not all cases are essential examples”.
7	Language grammar	−0.33	“Could the examples use passive voice without affecting the evaluation of the OER?” “It should be considered the active and passive part of the grammar, however, and based on my experience it seems to me that the adequacy of the language is the one that responds to the understanding of the content that is why I find it useful but not essential”.
8	Inclusive language	0	“I would define integrating language as a language without discrimination, considering covering more possibilities (gender, skin color, ethnic origin, language, religion, disability, etc.)”. “Generally speaking, a general language is used to simplify the way to name the subjects, without labeling, however there may be language that is offensive to some people, that is why it must be included, although not in an essential way”.
33	Video (duration)	0	“According to Coursera’s studies, the platform that offers its courses in video format recommends that the duration of the video be a minimum of 6 min and a maximum of 12, so that the user’s attention is active and dynamic, without boredom falling”.

The comments are shown in a question open to comments for each criterion.
